# Strengthening the United Nations Secretary-General’s Mechanism to an alleged use of bioweapons through a quality-assured laboratory response

**DOI:** 10.1038/s41467-021-23296-5

**Published:** 2021-05-25

**Authors:** Sandra Appelt, Anna-Maria Rohleder, Cédric Invernizzi, Robert Mikulak, Annika Brinkmann, Andreas Nitsche, Maren Krüger, Martin B. Dorner, Brigitte G. Dorner, Holger C. Scholz, Roland Grunow

**Affiliations:** 1 Centre for Biological Threats and Special Pathogens (ZBS 1, ZBS 2 and ZBS 3), Robert Koch Institute, Berlin, Germany; 2 Spiez Laboratory, Spiez, Switzerland; 3 Biological Policy Staff, Bureau of International Security and Nonproliferation, U.S. Department of State, Washington, USA

**Keywords:** Synthetic biology, Institutions

## Abstract

The cascade of innovations in biotechnology opens new pathways for biological warfare. The international laboratory network being developed under the UN Secretary-General’s Mechanism could provide vital evidence in case of an alleged biological attack.

## Comment

**Fig. 1:**
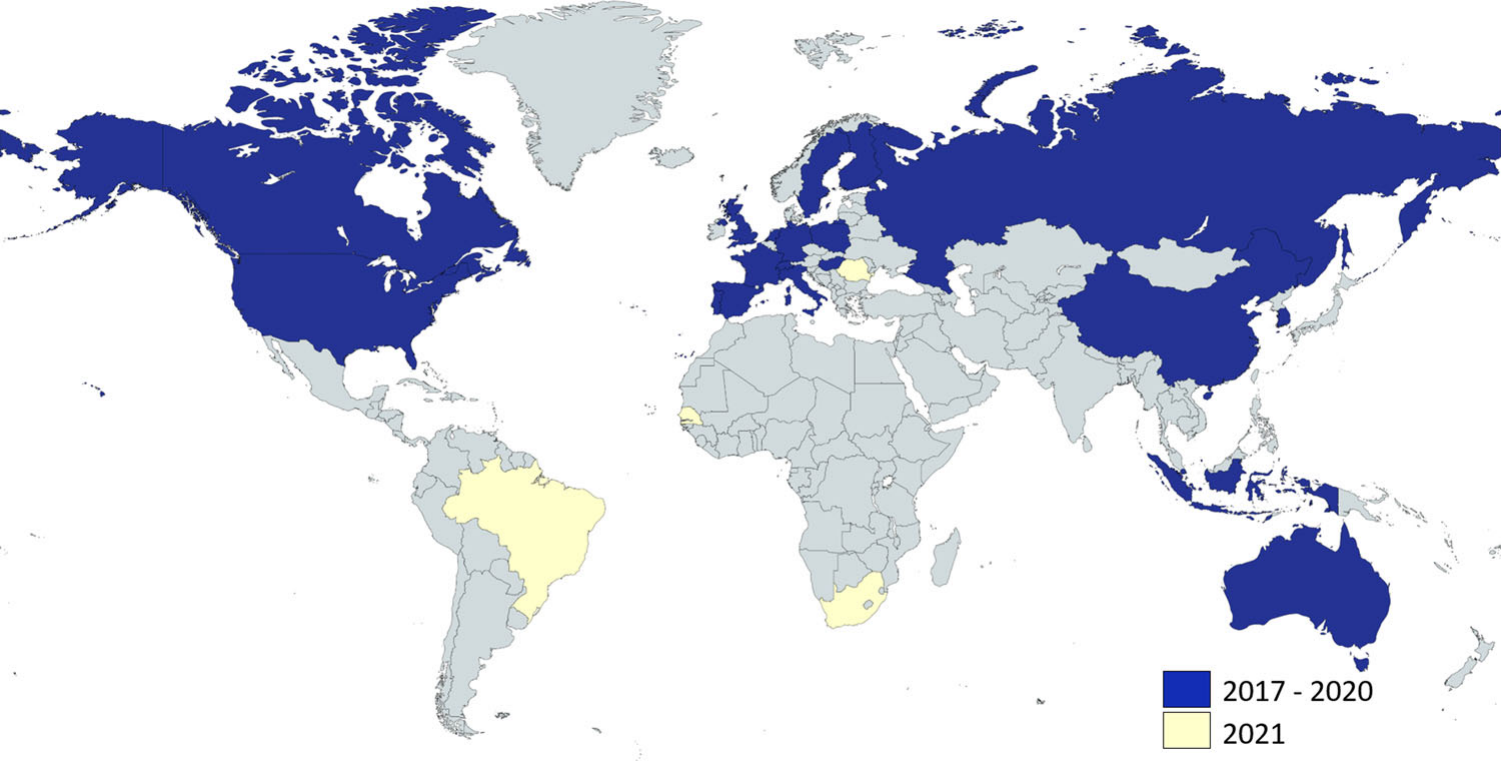
Global distribution of laboratories participating in RefBio. Highlighted in blue are laboratories from UN Member States already involved, highlighted in yellow are laboratories interested in future participation. The figure was created with mapchart.net licensed under a Creative Commons Attribution-ShareAlike 4.0 International License.

Inspired by Gregory Lewis’ very significant article ‘The biosecurity benefits of genetic engineering attribution’^1^, we want to emphasize a specific aspect: the importance of demonstrated and globally distributed capabilities to investigate possible deliberate misuse of technical innovation to engineer biological weapons. This aspect is a key feature of a recently established laboratory network (Fig. 1) supporting the United Nations in investigating alleged biological attacks by providing diagnostic analysis and forensic evidence.

Although the 1975 Biological and Toxin Weapons Convention provides a complete ban on biological weapons, a significant security threat remains. Serious violations of the Convention have occurred, and concerns also exist about on-going activities. In addition, terrorists and other non-state actors have sought or expressed the intention to use biological weapons.

As demonstrated at the beginning of the 21st century, when the anthrax attacks in the United States were investigated^2,3^, the threat from biological agents used as weapons has become more diverse and complex: this includes genetic engineering techniques, developed for peaceful purposes, and an easier availability of dangerous agents, as already discussed for Ebola^4^. Biotechnical advances have led to options to increase the contagiousness of agents, i.e., to render bacteria more virulent or insensitive to treatment^5^.

The public health and security risks posed by possible misuse of new biological techniques make it necessary to develop multi-sectoral biosecurity counterstrategies and to assess the “dual-use” potential of any relevant experiment^6–8^. These risks will be reduced by implementing efficient response strategies^9^ and investigative measures, starting with tailored laboratory diagnostics and forensic profiling to close attribution gaps. Any attribution of responsibility for an alleged use of biological weapons must be credible and well-founded, as the legal and political consequences are far-reaching. Under these circumstances, it needs to be considered that perpetrators might rather want to claim responsibility for an attack than obfuscate it and, thus, might seek to manipulate correct attribution^1^.

When examining the origins of a disease outbreak and determining whether the sources were natural or man-made, it is crucial that experimental results are made watertight for the legal system to be able to attribute source and perpetrator, as well as for decision-makers to take appropriate actions, recognizing that attributing responsibility is highly sensitive. Few algorithms are publicly available to technically address this question^10,11^. Although Next Generation Sequencing (NGS) approaches allow to obtain an immense amount of information, complex samples still require the initial detection and characterization of the causative agent. Whenever possible, the sample analysis should begin with isolation and enrichment of the pathogen. To permit a reliable sample analysis, the accompanying flora needs to be removed but should be kept for forensic evidence.

In the case of an alleged use of bioweapons, the UN Secretary-General (SG) may launch an international investigation, relying on a roster of expert consultants, qualified experts, and analytical laboratories nominated by the Member States. The elements of the so-called UN Secretary-General’s Mechanism are summarized in Table 1. The UNSGM operates under the umbrella of the United Nations Office for Disarmament Affairs (UNODA). Before 2017, little information was available on technical capabilities of nominated laboratories to select the most suitable in case of an alleged biological attack. To fill this gap, “RefBio” (“Germany’s contribution to strengthen the bio-analytical reference laboratories in the UNSGM”) was initiated with the aim to get a better overview of capabilities, as well as to increase expert knowledge and diagnostic skills. The project is strongly embedded in multilateral efforts to strengthen the UNSGM’s operational capabilities that have been successfully pursued by UNODA and UN Member States, including Australia, Denmark, France, Germany, Sweden, Switzerland, the United Kingdom, and the United States of America. Additional UNSGM-related exercises in bioinformatics have also been conducted in projects sponsored by the United States.

**Table 1:** Functions in the United Nations Secretary-General’s Mechanism (UNSGM) as described in the ‘Guidelines and Procedures for the Timely and Efficient Investigation of Reports of the Possible Use of Chemical and Bacteriological (Biological) or Toxin Weapons’^14^.

Role	Function in the UNSGM
Secretary-General (SG)	Launches investigations in case of an alleged use of a biological weapon on request of a UN Member State.
United Nations Office for Disarmament Affairs (UNODA)	Custodian of the UNSGM.
Expert consultant (nominated by the UN Member States)	Advises the SG on all issues during the entire investigation.
Qualified expert (nominated by the UN Member States; selected by the SG and expert consultants for the on-site mission team)	Is part of the on-site mission team; responsible for *inter alia*: -sample-taking according to established guidelines and procedures, -preliminary on-site tests, - shipment of samples to analytical laboratories, - preparation of the investigation report to be submitted to the SG.
**Analytical laboratories (nominated by the UN Member States)**	Are selected for UNSGM missions depending on tasks and agents; are asked to: - analyze the samples collected by the mission team to confirm the agent causing the outbreak; - collect analytical evidence pertaining to an alleged use of a bioweapon. Should fulfill at least the following requirements: - availability of high containment facility, - validated methods, including in-depth characterization and effective procedures to avoid cross-contamination, - robust quality management system, including security procedures for data and material protection, - capability to culture the agents in question, - demonstrated expertise in handling and analyzing potential bioterrorist agents, - capability to receive infectious material

Laboratories participating in RefBio need to fulfil specific technical requirements (Table 1) which are regularly tested and trained in tailored exercises simulating real-life investigations. Together with the test material, the participants are offered fictitious, but realistic, scenarios: typically, after an outbreak, UN Member State A alleges that State B has used agent X as a bioweapon. The SG is requested to investigate and to report on any findings on the origin of the outbreak, whether natural, accidental, or deliberate.

To correlate with the scenario, food, environmental, or clinical samples are mimicked. Possible threat agents (e.g., *Yersinia pestis*, *Bacillus anthracis*, orthopoxviruses, or ricin) are spiked into a variety of matrices. The exercises consist of two parts with different levels of difficulty: the first part serves the identification or exclusion of the suspected agent, whereas the second part requests laboratories to conduct deep molecular analyses and a profound characterization of the agent, including e.g., taxonomic classification, search for virulence genes, antibiotic resistance genes for bacteria, and further specific information hinting at a deliberate release of the agent.

The sequence analysis capabilities are considered a central part of the exercises. Any genetic element that is out of place requires particular attention when searching for genetic manipulation. Site-directed or insertional mutagenesis can be identified by detecting foreign or extrachromosomal DNA elements in cells which lead to gain or loss of specific functions (increased reproduction rate, altered host range). The status of DNA methylation is known to reveal information on possible genetic engineering. The analysis of single-nucleotide polymorphisms (SNP) on the level of core genomes may serve to unveil possible attribution of microbes’ origin and to establish attribution links. However, it may be hard to discriminate natural from deliberate modification of DNA or proteins. In this context, directed evolution techniques, imitating natural selection to activate mechanisms of mutagenesis, should be considered, too.

A high level of expertise is needed to identify genetic engineering and to reach conclusions about attribution. NGS is a widely used technology in this area, but also requires intricate bioinformatic skills. Extraction of suitable sample material, experimental setups (e.g., NGS platform, library preparation, PCR enrichment) and computational analysis (e.g., basecalling, assembly strategies, reference databases) come with their intrinsic limitations. An increased overall error rate as well as modified nucleotide substitution patterns influence downstream analyses (e.g., SNP calling). Consequently, the correct attribution of strains and source tracking is a challenge. Even high-quality sequences can yield false results when carrying out alignments to uncurated reference databases. Thus, laboratories that analyze samples during a UNSGM investigation need to have high-level capabilities in molecular forensics and a high level of awareness of the sum of all limitations to avoid over-interpretation of results. It would be advantageous to establish curated but openly accessible reference databases and to evaluate error rates in individual setups to predict the accuracy of results. This is of paramount importance to improve sequencing and to avoid artifacts that may lead to misinterpretation of results.

The project RefBio offers the laboratories an opportunity to train and exercise for a possible UNSGM mission, which is expected to facilitate a smooth, diligent, and comprehensive operation with added value at national and international level. During External Quality Assurance Exercises (EQAEs), the laboratories are asked to report their results to the organizer, just as in a real event. The individual results are evaluated and summarized in an overall report comprising all findings obtained, yet, in an anonymized manner. The EQAEs focus on the detection and characterization of selected biological agents and the identification of molecular “fingerprints” that may indicate or confirm an alleged use of a bioweapon. To reach an unambiguous identification of pathogens and biological toxins, different methodologies are to be applied^12,13^. Molecular forensics for biological toxins need to be further developed in cooperation with the Organization for the Prohibition of Chemical Weapons (OPCW), since biological toxins are also covered by the Chemical Weapons Convention.

Comparing the diagnostic performance of different laboratories, especially when it comes to the detection of molecular modifications, the need for further harmonization on the international level becomes apparent. Capabilities in molecular forensics need to be further developed to strengthen the attribution capabilities of the UNSGM. This includes also knowledge about natural genetic diversity and population structures of target microorganisms. Consequently, one goal of the RefBio project will be to provide suitable analytical tools and to allow access to up-to-date technologies, particularly in molecular typing and profiling of agents. Close collaboration and “twinning” of laboratories at different levels of capability are meant to facilitate the exchange of experience, knowledge, methods, and assays for mutual benefit. In such a sensitive area, trust is a fundamental prerequisite to closely collaborate among international partners. An overarching objective of RefBio is thus to establish a trusted international laboratory network with a broad geographical coverage.

Being aware of the omnipresent dual-use character of advances in biotechnology, we consider it a high priority task to develop international capabilities to assist in investigation of possible misuse. It is a demanding but inevitable challenge to tackle—the earlier, the better.

### Reporting summary

Further information on research design is available in the Nature Research Reporting Summary linked to this article.

## Supplementary information

Reporting Summary
